# Variation in Subjective Aging Among Middle-Aged and Older LGBTQ+ People: An Exploratory Study

**DOI:** 10.1093/geroni/igab046.2109

**Published:** 2021-12-17

**Authors:** Harry Barbee, Tara McKay

**Affiliations:** Vanderbilt University, Nashville, Tennessee, United States

## Abstract

Studies suggest that women and men have different experiences of subjective aging—including interpretations of age norms, timing of life course stages, and aging anxieties—but few have addressed variation within sexual and gender diverse communities. Drawing on a sample of middle-age and older LGBTQ+ people from Alabama, North Carolina, and Tennessee (n=923), we analyze how four dimensions of subjective aging (age-related self-perceptions, generalized views of aging, aging bodies, and aging anxieties) vary within the LGBTQ+ population by comparing gay and bisexual cisgender men (GBCM), lesbian and bisexual cisgender women (LBCW), and transgender, gender nonconforming, and non-binary (T/GNC/NB) people. Using multivariate regression models, we find that LBCW reported younger ideal ages, more elongated perceptions of the life course, more negative predictions of prospective health, and less aging anxiety compared to GBCM. Regarding self-perceptions and generalized views of aging, T/GNC/NB people reported younger ideal ages and more condensed perceptions of the life course compared to GBCM. Regarding perceptions of aging bodies, T/GNC/NB people reported more positive views of physical changes within the last five years and more negative predictions of prospective health compared to GBCM. Finally, T/GNC/NB people were less anxious than GBCM about future sex lives but were more anxious about not being able to support other people in the future. Overall, while some results align with studies of the general population, we find that sexual and gender diverse people may have different subjective aging trajectories, and thus experience differential physical and mental health outcomes, compared with cisgender heterosexual adults.

